# Mapping the landscape of human dopamine D2/3 receptors with [^11^C]raclopride

**DOI:** 10.1007/s00429-019-01938-1

**Published:** 2019-08-23

**Authors:** Goran Papenberg, Lars Jonasson, Nina Karalija, Jarkko Johansson, Ylva Köhncke, Alireza Salami, Micael Andersson, Jan Axelsson, Anders Wåhlin, Katrine Riklund, Ulman Lindenberger, Martin Lövdén, Lars Nyberg, Lars Bäckman

**Affiliations:** 1 Aging Research Center, Karolinska Institutet and Stockholm University, Tomtebodavägen 18A, 171 65 Solna, Sweden; 2 Umeå Center for Functional Brain Imaging (UFBI), Umeå University, Umeå, Sweden; 3 Department of Integrative Medical Biology, Umeå University, Umeå, Sweden; 4 Department of Radiation Sciences, Umeå University, Umeå, Sweden; 5 Center for Lifespan Psychology, Max Planck Institute for Human Development, Berlin, Germany; 6 Wallenberg Centre for Molecular Medicine, Umeå University, Umeå, Sweden; 7 Max Planck, UCL Centre for Computational Psychiatry and Ageing Research, Berlin, Germany; 8 Max Planck, UCL Centre for Computational Psychiatry and Ageing Research, London, UK

**Keywords:** [^11^C]raclopride, Dopamine D2/3 receptors, Inter-individual differences, Structural-equation modeling, COBRA study

## Abstract

The dopamine D2/3 system is fundamental for sensory, motor, emotional, and cognitive aspects of behavior. Small-scale human histopathological and animal studies show high density of D2/3 dopamine receptors (D2/3DR) in striatum, but also demonstrate the existence of such receptors across cortical and limbic regions. Assessment of D2/3DR BP_ND_ in the extrastriatal regions with [^11^C]raclopride has long been considered unreliable due to the relatively low density of D2/3DR outside the striatum. We describe the distribution and interregional links of D2/3DR availability measured with PET and [^11^C]raclopride across the human brain in a large sample (*N* = 176; age range 64–68 years). Structural equation modeling revealed that D2/3DR availability can be organized according to anatomical (nigrostriatal, mesolimbic, mesocortical) and functional (limbic, associative, sensorimotor) dopamine pathways. D2/3DR availability in corticolimbic functional subdivisions showed differential associations to corresponding striatal subdivisions, extending animal and pharmacological work. Our findings provide evidence on the dimensionality and organization of [^11^C]raclopride D2/3DR availability in the living human brain that conforms to known dopaminergic pathways.

**Electronic supplementary material:**

The online version of this article (10.1007/s00429-019-01938-1) contains supplementary material, which is available to authorized users.

## Introduction

Positron emission tomography (PET) can be used to quantify dopamine (DA) receptors in the human brain, using radioligands that bind selectively to the receptors of interest. [^11^C]raclopride is a well-validated tracer for assessment of striatal D2/3 DA receptor (D2/3DR) availability (or binding potential to non-displaceable tissue uptake; BP_ND_) (e.g., de Manzano et al. 2013; Egerton et al. 2010; Kim et al. 2014; Volkow et al. 2009). Competitive assay experiments in rhesus monkeys (Lidow et al. 1989) have documented that raclopride is a potent and selective D2/3DR ligand, which can be displaced from its binding sites only by D2/3DR-selective drugs in striatal and extrastriatal regions alike. Assessment of D2/3DR BP_ND_ in extrastriatal regions with [^11^C]raclopride has long been considered unreliable due to the relatively low density of D2/3DR outside the striatum (Hall et al. 1994; Farde et al. 1988). However, a study reported acceptable reliability for BP_ND_ based on [^11^C]raclopride in brain areas with low D2/3DR densities (*n* = 7; Alakurtti et al. 2015). More specifically, intraclass correlation coefficients (ICCs) for cortical areas indicated moderate to good reproducibility (e.g., temporal cortex: 0.79; dorsolateral prefrontal cortex: 0.86; superior and inferior frontal gyrus: 0.64 and 0.67). A more recent study reported similarly high ICCs across 7 months for [^11^C]raclopride BP_ND_ in both striatal and extrastriatal brain regions (*n* = 27; ICCs > 0.9 for frontal and temporal cortex; Karalija et al. 2019). This suggests that extrastriatal [^11^C]raclopride BP_ND_ values represent a true signal rather than mere noise. Here, we describe the ”landscape” of [^11^C]raclopride BP_ND_ in the human brain and investigate whether D2/3DR availability is organized according to anatomical and functional dopamine pathways, which would support the validity of extrastriatal [^11^C]raclopride measurements.

**Fig. 1:**
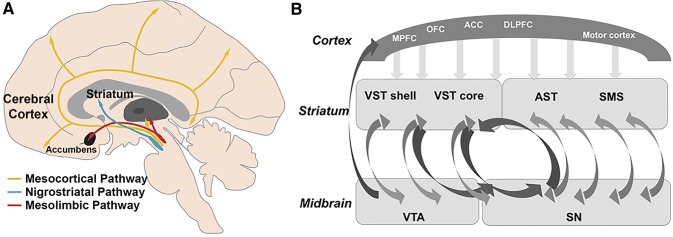
**a** Schematic illustration of the major dopaminergic pathways in the brain. Adapted from Li et al. (2009), with permission from Oxford University Press. **b** Schematic illustration of corticostriatal projections and spiral striato-midbrain–striatal pathways. *MPFC* medial prefrontal cortex, *OFC* orbitofrontal cortex, *ACC* anterior cingulate cortex, *DLPFC* dorsolateral prefrontal cortex, *VST* ventral striatum, *AST* associative striatum, *SMS* sensorimotor striatum, *VTA* ventral tegmental area, *SN* substantia nigra. Reprinted from Rieckmann et al. (2011), with permission from Oxford University Press

Dopaminergic projections, originating from the midbrain, densely innervate the striatum via the nigrostriatal pathway and, to a lesser degree, limbic and cortical areas via the mesolimbic and mesocortical pathways (Fig. 1a; Foote and Morrison 1987; Martinez et al. 2003; Haber and Knutson 2010; Cervenka et al. 2008). Striatum can also be functionally subdivided into limbic, associative, and sensorimotor parts based on corticostriatal projections (Foote and Morrison 1987; Haber and Knutson 2010; Tziortzi et al. 2014; Haber et al. 2006; Fig. 1b). The associative and sensorimotor pathways receive common dopaminergic innervation from the substantia nigra. The ventral tegmental area (VTA) provides dopaminergic input to the ventral striatum, as well as to limbic and neocortical areas via the mesolimbic and mesocortical pathways (Fig. 1a). With respect to the corticostriatal projections, the ventral striatum receives projections from the medial and orbitofrontal cortex, anterior cingulate cortex, as well as hippocampus and amygdala. The associative circuit involves dorsolateral prefrontal cortex, anterior cingulate cortex, and associative striatum (pre-commissural putamen and dorsal caudate nucleus). The anterior cingulate cortex is anatomically considered part of the limbic system, but is also connected to the associative cortices and projects to the associative striatum (Haber et al. 2006). Finally, the sensorimotor circuit encompasses the sensorimotor striatum (post-commissural part of dorsal putamen) and cortical areas, such as the precentral gyrus, postcentral gyrus, and superior parietal cortex.

We use structural equation modeling that accommodates the formation of latent factors that represent the common variance of their indicators. This method effectively separates out measurement error, and hence yields better estimates of factor variances and covariances (Little et al. 1999).We investigate between-person differences in D2/3DR availability across targeted brain regions belonging to anatomically defined pathways (i.e., striatal, limbic, neocortical). Given that corticostriatal projections impose a specific functional organization upon the striatum (Haber and McFarland 1999), we also examine whether individual differences in the [^11^C]raclopride BP_ND_ data support a functional subdivision of cortical areas, such that target regions belonging to the same functional loop also load on the same latent factor. A good fit for these models would support the use of [^11^C]raclopride BP_ND_ data to measure D2/3DR availability along known dopaminergic pathways across the brain. Moreover, we study whether the resulting functional corticostriatal factors are specifically related to their corresponding striatal targets.

## Materials and methods

The Cognition, Brain, and Aging (COBRA) study design, recruitment procedure, imaging protocols, and details of the cognitive and lifestyle battery have been reported elsewhere (Nevalainen et al. 2015). The study was approved by the local Ethical and Radiation Safety Committee of Umeå, Sweden, and all participants provided signed written informed consent prior to testing. Written consent was also acquired for storage of blood samples at Norrland’s University Hospital.

### Participants

The initial sample included 181 healthy older individuals (64–68 years of age; mean = 66.2; SD = 1.2; 81 women) who were randomly selected from the population register of Umeå, a city in northern Sweden. [^11^C]raclopride BP_ND_ data were excluded for four individuals with imperfect segmentation of MR images and PET–MR image coregistration and for one individual with pathological deviations in the brain observed on the MR images. Thus, the effective sample included 176 individuals.

### PET image acquisition

All participants underwent a PET scan (Discovery PET/CT 690; GE Healthcare) performed during resting-state conditions following an intravenous bolus injection of 250 MBq [^11^C]raclopride. Preceding the injection, a 5-min low-dose helical CT scan (20 mA, 120 kV, 0.8 s per revolution) was obtained for the purpose of PET-attenuation correction. Following the bolus injection, a 55-min 18-frame dynamic scan was acquired. Attenuation- and decay-corrected PET images (47 slices, field of view 25 cm, 256 × 256-pixel transaxial images, voxel size 0.977 × 0.977 × 3.27 mm^3^) were reconstructed with the iterative VUE Point HD-SharpIR algorithm (GE Healthcare); 6 iterations, 24 subsets, 3.0 mm postfiltering, yielding full width at half maximum of 3.2 mm (Wallsten et al. 2013). For comparative purposes, reconstruction was also performed with filtered-back projection (FBP; filter size: 6.4 mm). FBP is a reconstruction technique, which is often seen as a quantitative “gold standard” for larger regions. However, the image noise is rather high, which may cause FBP images to contain pixels with negative uptake values. Iterative techniques produce less noisy images, but converge at different rates for high and low uptakes. Thus, iterative techniques produce less noise, but at a possible cost of bias, especially at lower ranges (Walker et al. 2011; Jian et al. 2015; van Velden et al. 2009). Therefore, it is essential to validate extrastriatal findings with FBP reconstruction. Head movements during the imaging sessions were minimized with an individually fitted thermoplastic mask attached to the bed surface.

### PET data analyses

D2/3DR availability was determined by calculating [^11^C]raclopride BP_ND_ (Mintun et al. 1984; Innis et al. 2007; Logan et al. 1996). In brief, the PET emission scan format was converted from DICOM to NIfTI, corrected for head movements, and then coregistered to the corresponding MR image using the Statistical Parametric Mapping software (SPM8; Ashburner and Friston 2005). Regions of interest were delineated with the FreeSurfer 5.3 segmentation software (Han and Fischl 2007; Fischl et al. 2004; Fischl et al. 2002). Time–activity curves for striatal and extrastriatal regions and the cerebellum were used to calculate BP_ND_ using the Logan et al. (1996) graphical analysis, using perpendicular linear regression to minimize bias (Varga and Szabo 2002). The cerebellar gray matter was used as a reference region due to negligible D2/3DR expression (Camps et al. 1989; Farde et al. 1986; Levey et al. 1993). Median BP_ND_ data were extracted for all regions of interest based on the subcortical parcellations in FreeSurfer and the Desikan–Killiany atlas (Desikan et al. 2006) for extrastriatal regions. In addition, Brodmann areas 9 and 46 were defined based on masks from the MRIcron atlas (http://people.cas.sc.edu/rorden/mricron/index.html), as those are not available in the Desikan–Killiany atlas.

Notably, there are several possibilities in PET that could cause inflated extrastriatal BP or bias, all of which can be ruled out in our data set. First, bias could be introduced by iterative reconstruction methods, which is not present in FBP analysis. Toward this end, the correlations among brain regions are virtually identical in size when using FBP (see Table S1). A second possibility for a bias is noisy data in Logan analysis (Slifstein and Laruelle 2000). A method to remove such bias has been reported applying perpendicular linear regression (Varga and Szabo 2002), which was also done here before calculating BPs. Third, partial-volume effects due to the PET resolution may cause “spill-out” of radioactivity outside the high striatal uptake to nearby areas. The distance of this potential spill-out is related to the PET resolution, which for our scanner and iterative reconstruction algorithm was 3.2 mm radially and 4.7 mm axially full-width half maximum (Wallsten et al. 2013). This resolution may give appreciable spill-over at distances of the order of the resolution and this effect falls off quickly with distance, so that at four times the resolution (20 mm), no measurable effect is expected. Therefore, partial-volume effects are highly unlikely to cause measurable spillover from striatum to most cortical areas. That said, we cannot rule out some spillover to a few cortical regions, which are close enough. For instance, the most posterior part of the OFC may show some influence from the most anterior ventral striatum, and binding in the amygdala could impact adjacent parts of the temporal cortex.

A fourth origin of incorrect BP estimation and inflated correlations between regions has to do with reconstruction of projection data. Such effects are short range and also less of an issue with iterative reconstructions than with FBP (Razifar et al. 2005).

### Functional segmentation of striatum

To segment the striatum into its functional pathways (limbic, associative, sensorimotor), we followed the procedures described by Tziortzi et al. (2014), who performed tractography on a younger sample (aged 25–55 years). Instead of using the available masks from Tziortzi et al., we used the available segmentation results from one of our previous studies (*n* = 58; aged 64–78 years), where DTI data was acquired on the same scanner and the age range was more similar to the COBRA study (Jonasson et al. 2016). Therefore, we considered our seeds as being better suited for analyzing the functional subdivisions. Subject-specific striatal seeds were derived from FreeSurfer 5.3 (concatenated caudate, putamen, and nucleus accumbens), and Andri Tziortzi (GlaxoSmithKline) provided the three cortical target masks (limbic, associative, sensorimotor; see Tziortzi et al. (2014), for definition of masks).In short, using the FMRIB’s diffusion toolbox (http://www.fmrib.ox.ac.uk/fsl/), a model estimating crossing fibers within voxels was run on eddy current corrected and betted diffusion weighted volumes (Behrens et al. 2007). Fibers originating from the striatum were tracked until they terminated in either of the three cortical targets. On the subject level, each striatal voxel was assigned to the cortical target to which it had the most terminating fibers to produce a subject-specific functional segmentation. This segmentation was then transformed to standard space by applying a transform created by normalizing a T1-weighted scan to the 1 mm non-linear MNI template using FMRIB’s non-linear registration toolbox (FNIRT). In standard space, each striatal voxel was assigned to the functional target to which most subjects had their corresponding voxels assigned.

### Statistical analyses

One-sample *t* tests were conducted to determine whether D2/3DR availability were reliably greater than zero, particularly for extrastriatal regions. To facilitate comparability, we describe mean [^11^C]raclopride BP_ND_ for the same regions of interest as used by Hall et al. (1994), who quantified D2/3DR availability in six post-mortem brains using [^3^H]raclopride. We directly compare their measure of receptor density (i.e., *B*_max_) with our data of BP_ND_ at the group level, using Spearman’s correlations. Values for *B*_max_ based on [^3^H]raclopride from Hall et al. (Fig. 1b in the original publication) were digitized with the PlotDigitizer software (http://plotdigitizer.sourceforge.net) and are reported in the supplementary (Table S2). Moreover, we also compared regional [^11^C]raclopride BP_ND_ values with values determined with the high-affinity ligand [^18^F]fallypride (Seaman et al. 2019; https://osf.io/h67k4/). [^18^F]fallypride data were obtained from older adults with a similar age range as in the COBRA study (aged 60–67, *n* = 17).

**Fig. 2:**
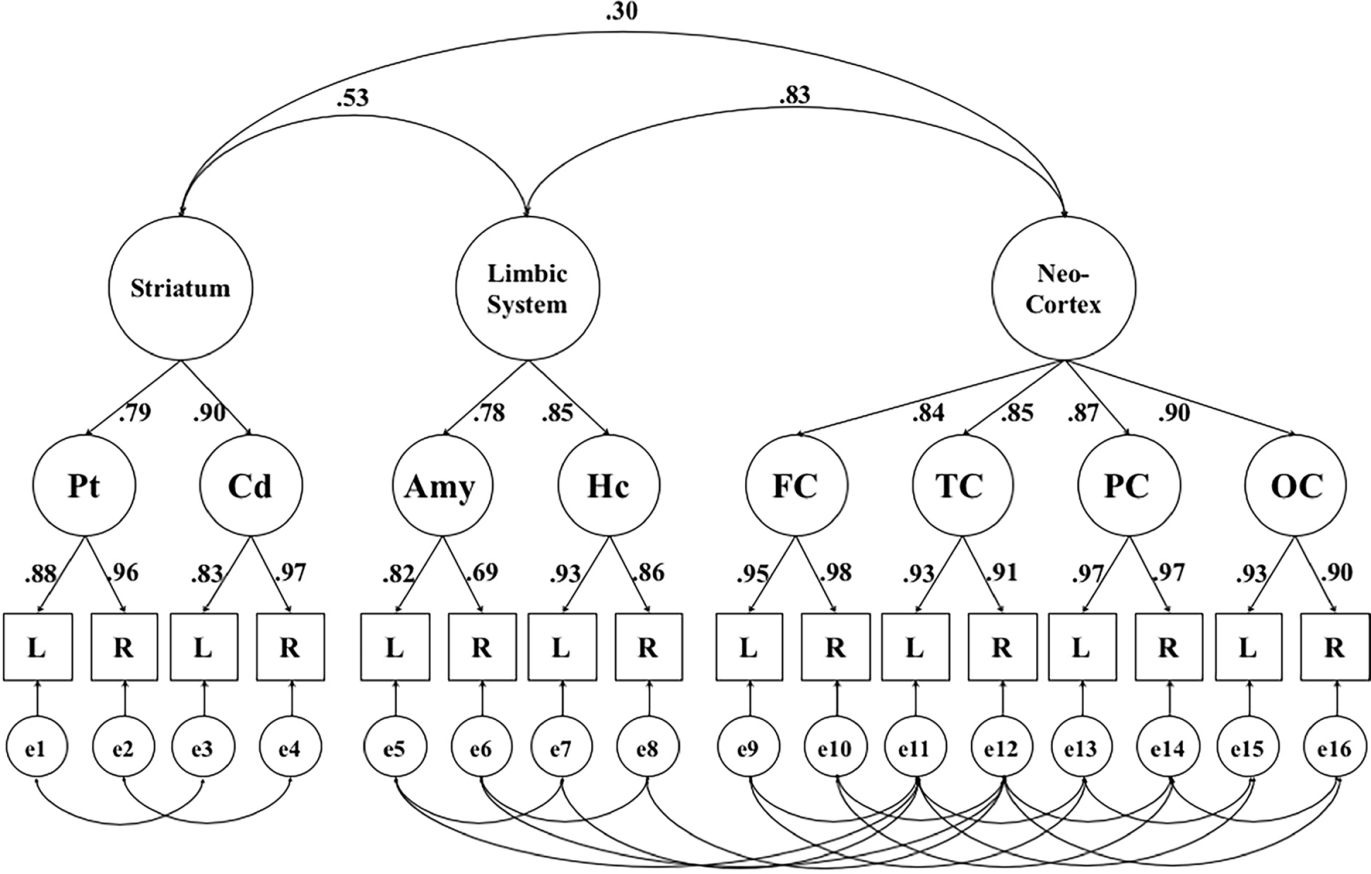
Hierarchical factor model portraying the relationship between [^11^C]raclopride D2/3DR BP_ND_ in striatum, limbic system, and neocortex. The figure shows standardized factor loadings and factor correlations for this model. *Pt* putamen, *Cd* caudate, *Hc* hippocampus, *Amy* amygdala, *FC* frontal cortex, *OC* occipital cortex, *TC* temporal cortex, *PC* parietal cortex, *L* left hemisphere, *R* right hemisphere, *e* error. Errors represent hemisphere-specific variance and hemisphere-specific measurement error. For any indicator, the variance accounted for by its error term corresponds to one minus the square of its factor loading

**Fig. 3:**
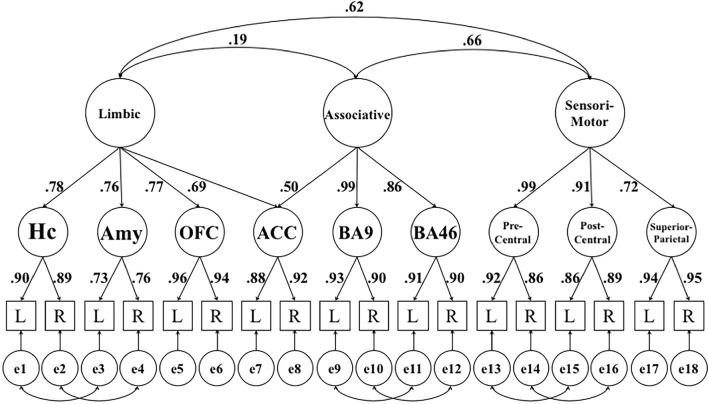
Hierarchical factor model reflecting interrelations of [^11^C]raclopride D2/3DR BP_ND_ between functional subdivisions in extrastriatal regions. The figure shows standardized factor loadings and factor correlations for this model. *Hc* hippocampus, *Amy* amygdala, *OFC* orbito-frontal cortex (lateral, medial), *ACC* anterior cingulate cortex, *BA9* Brodmann Area 9, *BA46* Brodmann Area 46, *Precentral* precentral gyrus, *Postcentral* postcentral gyrus, *Superior parietal* superior parietal lobule, *L* left hemisphere, *R* right hemisphere, *e* error. Errors represent hemisphere-specific variance and hemisphere-specific measurement error. For any indicator, the variance accounted for by its error term corresponds to one minus the square of its factor loading

Further analyses were conducted within the structural equation modeling framework, using AMOS 7.0 (Arbuckle 2006). We estimated two hierarchical models. Whereas the anatomical model explored the factor structure among striatum, limbic system, and neocortex (Fig. 2), the second model postulated that the functional subdivision of the limbic system and neocortex was a good representation of the BP_ND_ data (Fig. 3).

Structural-equation modeling combines confirmatory factor analysis and multiple regression or covariance analysis, accommodating the specification of a theory-based statistical model. The confirmatory factor model part enables defining latent factors based on measured variables. One of the advantages of structural-equation models (SEM) is that latent factors are considered free of error. All variance common to the observed variables will be represented in their latent factor and all remaining variance is estimated as error (i.e., residual) variance. The regression or covariance part enables examining relationships among latent factors. Both parts are specified in a single model and the entire model is fitted to the data simultaneously. That is, variances and covariances of all variables in the data are compared to the variances and covariances implied by the model. A number of well-established fit indices are used to evaluate how well the model fits the data. If a specific model does not represent the data well, then this will be reflected in unacceptable fit indices and should be rejected (for further information on the use of SEM in cognitive neuroscience, see Kievit et al. 2017).

Following standard notation (Boker et al. 2009), in Figs. 2 and 3 boxes indicate observed variables, circles represent latent factors, arrows denote factor loadings, and double-headed arrows indicate correlations. In both models, [^11^C]raclopride BP_ND_ of each region of interest (ROI) in the left and right hemisphere were used to reflect first-order latent factors, extracting the common variance across hemispheres (Raz et al. 2005). The second-order factors represent variance that is common to the first-order factors. To define a metric for the factors, one loading on each factor was fixed to 1. To estimate latent means for the best fitting models, the intercept of the scaling indicators of each first-order factor was fixed to zero, providing a scale for the latent mean. Indicators for neocortical latent factors in Fig. 2 are unit-weighted composite scores based on all available regions in the Desikan–Killiany atlas for the frontal, temporal, occipital, and parietal cortex (Desikan et al. 2006). For comparative purposes, we also fitted an alternative model positing that common variance across the indicators (Fig. 2) generalizes across the brain, so that all indicators load on one general factor (Figure S1).

To reduce model complexity for the functional subdivisions and have a comparable number of regions of interest across functional loops (Fig. 3), we focused on a limited number of target regions within the limbic (hippocampus, amygdala, orbito-frontal cortex, anterior cingulate cortex), associative (anterior cingulate cortex, Brodmann areas 9 and 46), and sensorimotor (precentral gyrus, postcentral gyrus, superior parietal cortex) regions. Given that the anterior cingulate cortex (ACC) is associated with both the limbic and associative loops, as described above, its first-order latent factor loads on both the limbic and associative factor. Again, for comparative purposes, we fitted an alternative model positing that common variance across the indicators (Fig. 3) generalizes across the brain, so that all indicators load on one general factor (Figure S1).

In the next step, the latent factors of the corticolimbic subdivisions were related to the corresponding BP_ND_ values for ventral, associative, and sensorimotor striatum. Whenever a particular striatal subdivision of interest was associated with multiple corticolimbic factors, we report associations adjusted for the other two subdivisions to determine the unique links. Residual variances, which are variances not explained by the latent factors, were allowed to covary between neighboring brain areas in all models (Figs. 2, 3). We evaluated whether the models provided a good representation of the data, using the root mean square error of approximation (RMSEA) and the comparative fit index [CFI; see Kline 2005, for interpretation of these indices)]. Values ≤ 0.08 for RMSEA and ≥ 0.90 for CFI were considered to indicate good model fit.

Both univariate (± 3.29 SD) and multivariate outliers (Mahalanobi’s distance; *p* < 0.001 threshold for the *χ*^2^ value) were excluded from analyses and treated as missing by the program (< 1% of values; Tabachnick and Fidell 2006). All variables displayed acceptable skewness and kurtosis (i.e., values not exceeding ± 1.5). In all analyses, the alpha level for statistical decisions was set to 0.05.

## Results

### Landscape of D2/3DR availability in neocortical and subcortical areas

**Fig. 4:**
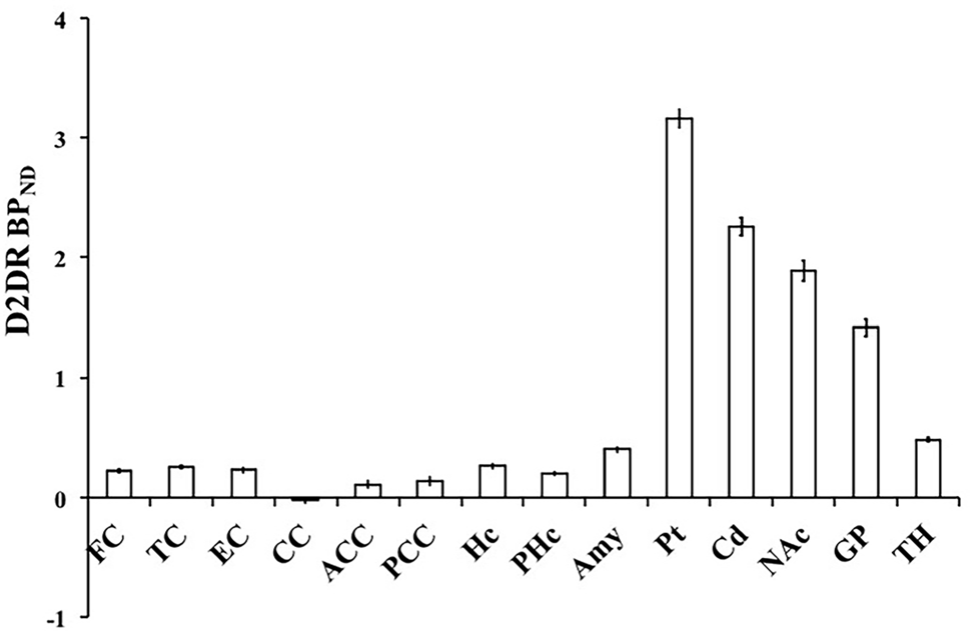
Mean [^11^C]raclopride D2/3DR BP_ND_ across brain regions. *FC* frontal cortex (superior, medial, oribital), *TC* temporal cortex (superior, middle), *EC* entorhinal cortex, *CC* corpus callosum, *ACC* anterior cingulate cortex, *PCC* posterior cingulate cortex, *Hc* hippocampus, *PHc* parahippocampus, *Amy* amygdala, *Pt* putamen, *Cd* caudate, *NAc* nucleus accumbens, *GP* globus pallidus, *TH* thalamus. Error bars represent 95% confidence intervals around the means

**Fig. 5:**
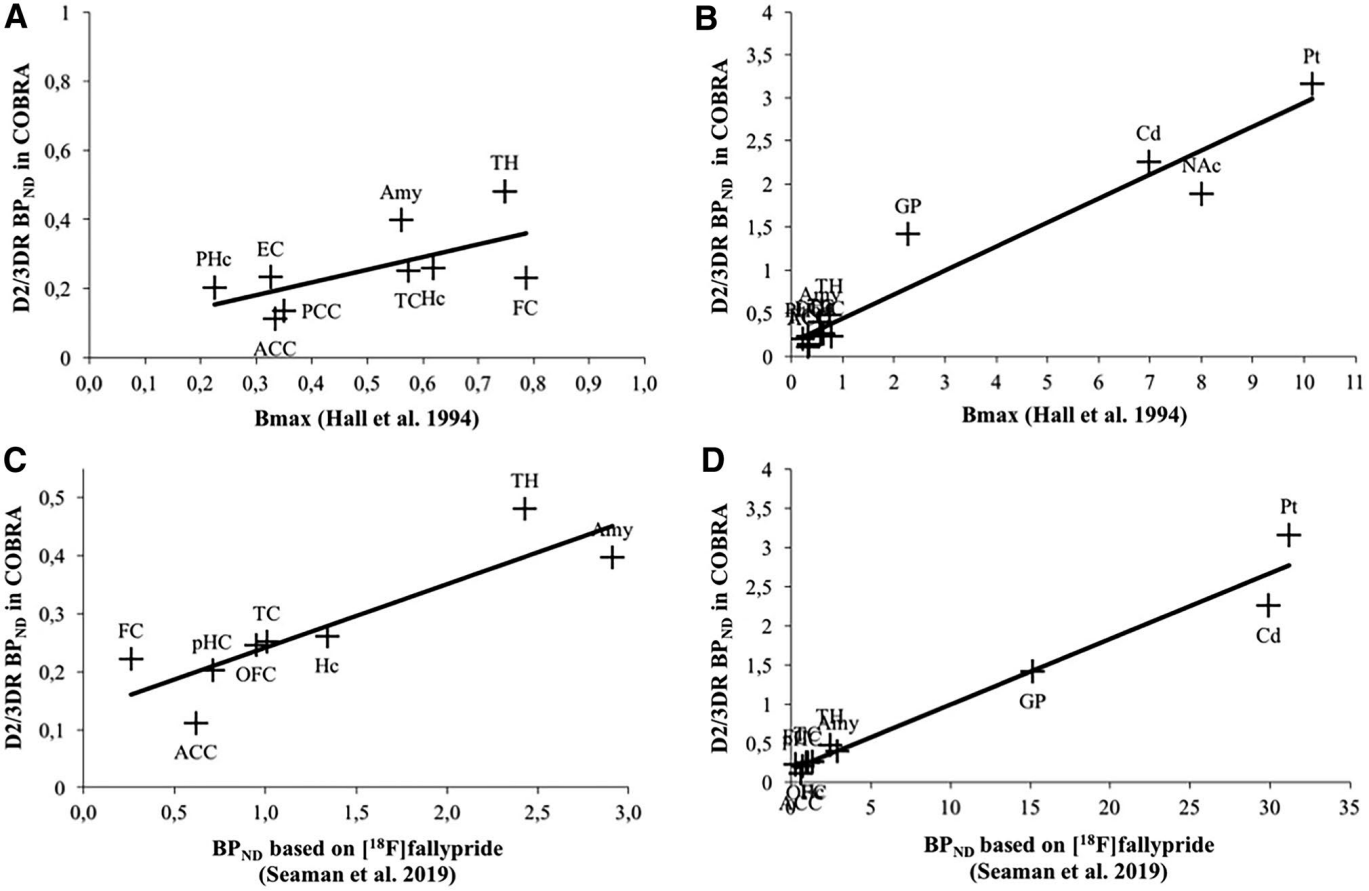
Relationship between [^11^C]raclopride D2/3DR BP_ND_ in COBRA and receptor density (*B*_max_) in post-mortem brains as reported by Hall et al. (1994) across extrastriatal regions of interest (**a**) and including the striatum and globus pallidus (**b**). Relationship between [^11^C]raclopride D2/3DR BP_ND_ in COBRA and BP_ND_ based on [^18^F]fallypride (Seaman et al. 2019) across extrastriatal regions of interest (**c**) and including the striatum and globus pallidus (**d**). *FC* frontal cortex (*Hall* superior, medial, orbital in **a** and **b**, *Seaman* middle, superior, and inferior frontal gyrus, in **c** and **d**, *COBRA* superior frontal gyrus, rostral middle-frontal gyrus, medial orbital frontal cortex in **a** and **b** and superior frontal and rostral middle-frontal gyrus in **c** and **d**), *TC* temporal cortex (*Hall* superior and middle-temporal cortex in **a** and **b**, *Seaman* superior, middle, and inferior temporal gyrus in **c** and **d**, *COBRA* superior and middle-temporal gyrus); *EC* entorhinal cortex, *CC* corpus callosum, *ACC* anterior cingulate cortex, *OFC* orbitofrontal cortex (*Seaman*, anterior, medial, lateral, and posterior orbital gyrus, *COBRA* medial orbital frontal cortex), *PCC* posterior cingulate cortex, *Hc* hippocampus, *PHc* parahippocampus, *Amy* amygdala, *Pt* putamen, *Cd* caudate, *NAc* nucleus accumbens, *GP* globus pallidus, *TH* thalamus

Figure 4 depicts D2/3DR BP_ND_ values for striatal and extrastriatal regions (see Table S3 for specific values). All values, except for the corpus callosum, were reliably different from zero (*p*s < 0.05). The corresponding numbers for data based on FBP reconstruction were highly similar (Figure S2). Importantly, D2/3DR BP_ND_ correlates very highly with *B*_max_ reported by Hall et al. (Fig. 5b), supporting the validity of our data (Spearman’s correlation: *r* = 0.841; *p* = 0.000). The correlation was attenuated after excluding the striatum and globus pallidus (Spearman’s correlation: *r *= 0.533, *p* = 0.139; Fig. 5a). Note, however, that *B*_max_ values varied largely across frontal regions (Table S2 in supplementary). *B*_max_ of the superior frontal cortex was five times greater than *B*_max_ in the medial and three times greater than *B*_max_ in the orbitofrontal cortex, suggesting an overestimation of the superior frontal cortex. The very high *B*_max_ for the superior frontal cortex is also inconsistent with [^18^F]fallypride-PET data, which suggest similar levels of D2/3DRs across superior and medial frontal areas and relatively higher D2/3DR levels in orbital areas (Seaman et al., 2019). Excluding the superior frontal cortex revealed a strong correlation among regions with very low D2 density (Spearman’s correlation: *r* = 0.767, *p* = 0.016). In line with the post-mortem data, BP_ND_ based on [^11^C]raclopride correlated very highly with previously published [^18^F]fallypride BP_ND_ across extrastriatal regions (Spearman’s correlation: *r* = 0.907; *p* = 0.002; Fig. 5c), which was similar to the association obtained when including regions with high D2 density (Spearman’s correlation: *r* = 0.964; *p* = 0.000; Fig. 5d). Notably, the positive correlation between our BP_ND_ estimates and both post-mortem and [^18^F]fallypride data occur despite differences in ROI definition. Data from Seaman et al. are based on the Hammers atlas, whereas our ROI data are based on the Desikan–Killiany atlas.

Finally, Figures S3 and S4 illustrate similarities in time–activity curves and kinetics for regions with high (striatum) and low receptor density (frontal cortex), as compared to the reference region.

### Model including striatal, limbic, and neocortical factors

A model representing striatal, limbic, and neocortical second-order latent factors (Fig. 2) showed good fit, *χ*^2^ (75, *n* = 176) = 147.2, *p* < 0.05, CFI = 0.97, RMSEA = 0.074, CI_RMSEA_ (0.056, 0.092). Correlations among indicators for this model are also shown in Table S4. Standardized factor loadings of the indicators for the first-order factors were high, reflecting high correlations between hemispheres. Similarly, the first-order factors loaded highly on the second-order factors, indicating high correlations among hierarchically organized striatal, limbic, and neocortical regions. Finally, striatal D2/3DR BP_ND_ was positively correlated with neocortex (*r* = 0.30) and the limbic system (*r* = 0.53), although these two correlations could not be constrained to be equal without loss in fit (Δ*χ*^2^ (1, *n* = 176) = 18.6, *p* < 0.05). The inter-factor correlation between D2/3DR BP_ND_ in limbic system and neocortex (*r* = 0.83) was only significantly higher than the striatum–neocortex association (Δ*χ*^2^ (1, *n* = 176) = 12.2, *p* < 0.05). Constraining this correlation to 1 resulted in a significantly worse fit, Δ*χ*^2^ (1, *n* = 176) = 11.2, *p* < 0.05. This suggests that, despite the high correlation between the limbic system and neocortex, these two factors are separable. To test whether between-person differences generalize across the indicators, we also fitted a model in which all indicators load on one general factor. This model exhibited poor fit, *χ*^2^ (86, *n* = 176) = 1012.8, *p* < 0.05, CFI = 0.64, RMSEA = 0.248, CI_RMSEA_ (0.235, 0.262), suggesting that it represents the data inadequately (Table S5 for factor loadings). Latent means for first-order factors of the model shown in Fig. 2 are presented in Table 1.

**Table 1:** Latent mean estimates of the hierarchical factor model portraying the relationship between [^11^C]raclopride D2/3DR BP_ND_ in striatum, limbic system, and neocortex

ROI	Latent mean	SE
Pt	3.06	0.020
Cd	2.21	0.020
Amy	0.41	0.005
Hc	0.27	0.004
FC	0.19	0.003
TC	0.25	0.003
PC	0.20	0.003
OC	0.24	0.003

*ROI* region of interest, *Pt* putamen, *Cd* caudate, *Amy* amygdala, *Hc* hippocampus, *FC* frontal cortex, *TC* temporal cortex, *PC* parietal cortex, *OC* occipital cortex, *SE* standard error

All latent means are significantly different from zero at *p* < 0.001

### Functional subdivision of limbic and neocortical regions

A model postulating a functional subdivision of limbic and neocortical regions (Fig. 3) showed good fit, *χ*^2^ (116, *n* = 176) = 236.2, *p* < 0.05, CFI = 0.96, RMSEA = 0.077, CI_RMSEA_ (0.063, 0.091; see Table S6 for interrelationships among regions). Again, factor loadings for the first-order factors were high, reflecting high correlations between hemispheres. With respect to second-order factor loadings, ACC had equally high loadings on the limbic and associative factors, which is in line with its structural and functional associations in the brain (Haber et al. 2006). All other limbic, associative, and sensorimotor first-order factors loaded well on their respective second-order factors. Interestingly, the sensorimotor factor correlated similarly strongly with both the limbic (*r* = 0.62; *p* < 0.05) and associative (*r* = 0.66; *p* < 0.05) factors, whereas the relationship between the limbic and associative factors was significantly weaker (*r *= 0.19; *p* < 0.05), as indicated by a significant loss in fit after equating correlations (*p*s < 0.05 for Δ*χ*^2^). An alternative model positing that D2/3DR availability generalizes across functional DA pathways showed unacceptable fit, *χ*^2^ (129, *n* = 176) = 1247.3, *p* < 0.05, CFI = 0.61, RMSEA = 0.223, CI_RMSEA_ (0.211, 0.234), again suggesting that such a model represents the data inadequately (see Table S7 for factor loadings). Latent means for first-order factors of the model shown in Fig. 3 are presented in Table 2.

**Table 2:** Latent mean estimates of the hierarchical factor model reflecting interrelations of [^11^C]raclopride D2/3DR BP_ND_ between functional subdivisions in extrastriatal regions

ROI	Latent mean	SE
Hc	0.27	0.004
Amy	0.41	0.005
OFC	0.24	0.003
ACC	0.24	0.004
BA9	0.09	0.003
BA46	0.17	0.003
Precentral	0.15	0.003
Postcentral	0.11	0.003
Superior parietal	0.16	0.004

*ROI* region of interest, *Hc* hippocampus, *Amy* amygdala, *OFC* orbitofrontal cortex (lateral, medial), *ACC* anterior cingulate cortex, *BA9* Brodmann Area 9, *BA46* Brodmann Area 46, *Precentral* precentral gyrus, *Postcentral* postcentral gyrus, *Superior parietal* superior parietal lobule, *SE* standard error

All latent means are significantly different from zero at *p *< 0.001

Next, we correlated the latent factors (Fig. 3) with the functional segmentation of the striatum (ventral, associative, sensorimotor; mean of left and right). In line with the hypothesized functional subdivisions, BP_ND_ in the ventral striatum was positively related to BP_ND_ for the cortical limbic factor (*r* = 0.27; *p* < 0.05), whereas the relations to the associative (*r* = − 0.02; n.s.) and sensorimotor (*r* = − 0.05; n.s.) factors were not significant. Likewise, the associative striatum correlated positively with the associative factor (*r* = 0.30; *p *< 0.05), after adjusting for the limbic and sensorimotor striatum. The correlations with the limbic (*r* = − 0.14; n.s.) and sensorimotor (*r* = 0.01; n.s.) factors were not reliable. Significant and non-significant correlations were reliably different from each other (*p* < 0.05 for Chi square difference tests, after equating correlations pairwise). Finally, the sensorimotor striatum correlated positively with the sensorimotor factor (*r* = 0.28; *p* < 0.05), after adjusting for the ventral and associative striatum. In this case, a positive, albeit weaker, correlation was also observed with the limbic factor (*r* = 0.21; *p* < 0.05), whereas the association with the associative factor was not significant (*r *= 0.10; n.s.). Notably, the association with the sensorimotor factor was not reliably different from that with the limbic factor, but differed from the link to the associative factor (*p* < 0.05 for Chi square difference tests). All critical correlations survived Bonferroni correction for nine comparisons (*p *= 0.006).

## Discussion

We investigated the distribution and interregional associations of [^11^C]raclopride BP_ND_ across the brain in a sample of 176 older adults. Between-person differences in D2/3DR availability measured with [^11^C]raclopride could be accounted for by an anatomical model (striatum, limbic system, and neocortex) as shown previously with the high-affinity ligand [^18^F]fallypride (Zald et al. 2010). Importantly, we extended that study by showing that the data are organized according to known dopaminergic pathways across the striatum and cortex (limbic, associative, and sensorimotor), and by showing specific associations between the corresponding striatal and cortical functional divisions.

The use of [^11^C]raclopride to measure extrastriatal D2/3DR availability has long been considered unreliable due to the low density of D2/3DR (Farde et al. 1986). However, recent studies provided evidence of good test–retest reliability for raclopride in extrastriatal regions (Alakurtti et al. 2015; Karalija et al. 2019). There are several indications why [^11^C]raclopride is suitable for measuring the distribution of D2/3DR across the brain. First, we show that [^11^C]raclopride BP_ND_ in extrastriatal regions is correlated with post-mortem (Hall et al. 1994) and [^18^F]fallypride estimates of D2/3DR receptor density. Second, the SEM analyses further support both reliability and validity of [^11^C]raclopride in measuring extrastriatal D2/3DR BP_ND_. The good model fit indicates both convergent and discriminant validity, as the organization of [^11^C]raclopride BP_ND_ is in line with anatomical and functional DA pathways. Convergent validity is supported by the higher associations among regions that should, according to theory, correlate with each other (i.e., the strongest correlations were found between hemispheres and within DA pathways). At the same time, discriminant validity is supported by relatively weaker associations among regions that, according to theory, should be less strongly correlated. Further, the fact that the left and right hemisphere show such strong correlations, resulting in very high first-order factor loadings (around 0.9) suggests that there is a lot of shared variance, supporting the reliability of the measurements. Thus, overall, our findings demonstrate that extrastriatal D2/3DR estimation with [^11^C]raclopride is associated with measures and patterns that result from target binding, rather than represent noise.

Our SEM analyses demonstrate good fit for a model assuming separate factors for striatal, limbic, and neocortical BP_ND_, which are positively correlated. The link between the limbic system and neocortex was particularly high, which may be due to both being innervated by the VTA via the mesocortical and mesolimbic pathways (Fig. 1a). Importantly, our data are in line with a study using the high-affinity ligand [^18^F]fallypride (Zald et al. 2010) to investigate interindividual differences in D2/3DR availability: Individual differences in D2-like BP_ND_ were accounted for by three distinguishable factors, representing striatal, neocortical, and limbic regions. Moreover, only 10% of the variance in the overall cortical D2-like BP_ND_ was accounted for by striatal D2-like BP_ND_, which is almost identical to the current pattern of data (9% of explained variance). Thus, our conclusions regarding the anatomical factor structure based on extrastriatal [^11^C]raclopride data are highly similar to those derived from [^18^F]fallypride data. Whereas our study is well powered and supports the reliability and validity of extrastriatal D2/3DR assessments with [^11^C]raclopride, high-affinity ligands such as [^11^C]-FLB457 (Halldin et al. 1995) and [^18^F]fallypride (Mukherjee et al. 2004) may still be more beneficial for extrastriatal D2/3DR assessments in studies with small sample sizes due to higher signal-to-noise ratios.

Furthermore, the structure of individual differences suggests that neocortical and limbic BP_ND_ data are organized in accordance with functional subdivisions of the DA system (limbic, associative, sensorimotor). The association between the limbic and associative factors was reliably lower than that between the associative and sensorimotor factors. This likely reflects common dopaminergic innervation from the substantia nigra for the latter two cortical subdivisions (Martinez et al. 2003; Rieckmann et al. 2011). By contrast, mesolimbic and mesocortical areas both receive input from the VTA (Fig. 1). The functional subdivision pertaining to the corticolimbic BP_ND_ data is further supported by the specificity of correlations for the limbic and associative factors with the corresponding striatal subdivisions. For the sensorimotor striatum, positive associations were observed with both sensorimotor and limbic regions. The latent correlations between the limbic and sensorimotor factors were high as well. Toward this end, a PET imaging study showed that administration of amphetamine induces larger reduction in D2/3DR availability in the ventral and sensorimotor striatum compared to associative regions (Martinez et al. 2003). As noted by Trifilieff and Martinez (2014), the sensorimotor striatum shares histochemical features with the ventral striatum (Fudge and Haber 2002) and receives glutamatergic input from amygdala and other limbic regions (Fudge and Haber 2002; Fudge et al. 2004). This suggests that the sensorimotor striatum may also involve a limbic component, as reflected by the observed associations between D2/3DR BP_ND_ in the sensorimotor striatum and corticolimbic areas.

DA-PET studies are typically characterized by relatively small sample sizes, which preclude examining interregional correlations of BP_ND_ values in latent space. The observed interindividual differences in anatomical and functional DA pathways in the current sample likely originate from genetic influences that are further augmented by recursive relations between epigenetic and environmental factors that operate across the lifespan (Beam and Turkheimer 2013). Research shows that different interventions, such as cognitive training and physical exercise, can have selective effects on different parts of the DA system. For example, exercise training across 8 weeks in methamphetamine users increased their D2/3DR binding in striatum, with no effects in extrastriatal regions (Robertson et al. 2015). An intervention study targeting D2/3DR demonstrated increased striatal DA release following 5 weeks of working-memory training (Bäckman et al. 2011). Furthermore, candidate gene studies have reported that variations in the same single-nucleotide polymorphism, located in the D2 gene, influence D2/3DR BP_ND_ differently in striatal (Hirvonen et al. 2009a) and extrastriatal (Hirvonen et al. 2009b) brain areas. These genetic effects may become magnified in aging (Papenberg et al. 2015), contributing to between-person differences in D2/3DR availability across the brain. Finally, the SEM approach enables studying D2/3DR distributions within and between anatomical and functional DA pathways. The novel models proposed may prove useful when investigating selective decline within and across pathways associated with the discussed genetic and lifestyle factors, but also in healthy aging (Bäckman et al. 2006) and neurological disorders (Heckman et al. 2016).

Collectively, our findings provide evidence on the dimensionality and organization of D2/3DR availability in the living human brain and support the reliability and validity of whole-brain measurements of D2/3DR availability with [^11^C]raclopride.

## Electronic supplementary material

Below is the link to the electronic supplementary material.

Supplementary material 1 (DOCX 261 kb)

## Data Availability

The data sets generated and analyzed during the current study are available from the corresponding author on reasonable request.
