# Supplementary material for: Mapping the landscape of human dopamine D2/3 receptors with [11C]raclopride
Source: Brain Struct Funct. 2019 Aug 23;224(8):2871–82. doi: 10.1007/s00429-019-01938-1 (PMC6778542; doi:10.1007/s00429-019-01938-1)
Supplement: Supplementary file 1 — Supplementary material 1 (DOCX 261 kb) [file 429_2019_1938_MOESM1_ESM.docx]

**Appendix**


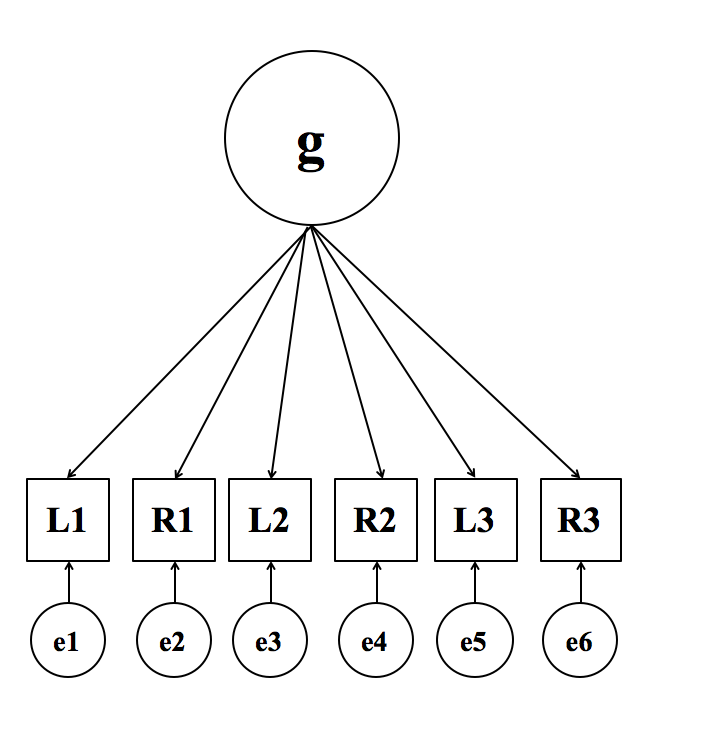


*Figure S1*. Schematic representation of an alternative model positing that common variance across the indicators generalizes across the brain, in this example with three regions of interest. The specific indicators and factor loadings of the alternative models are presented in Table S5 and S7. Residual variances, which are variances not explained by the latent factors, were allowed to covary between neighboring brain areas as in the hierarchical models (see Figures 2 & 3); L = Left Hemisphere; R = Right Hemisphere; e = error.

*
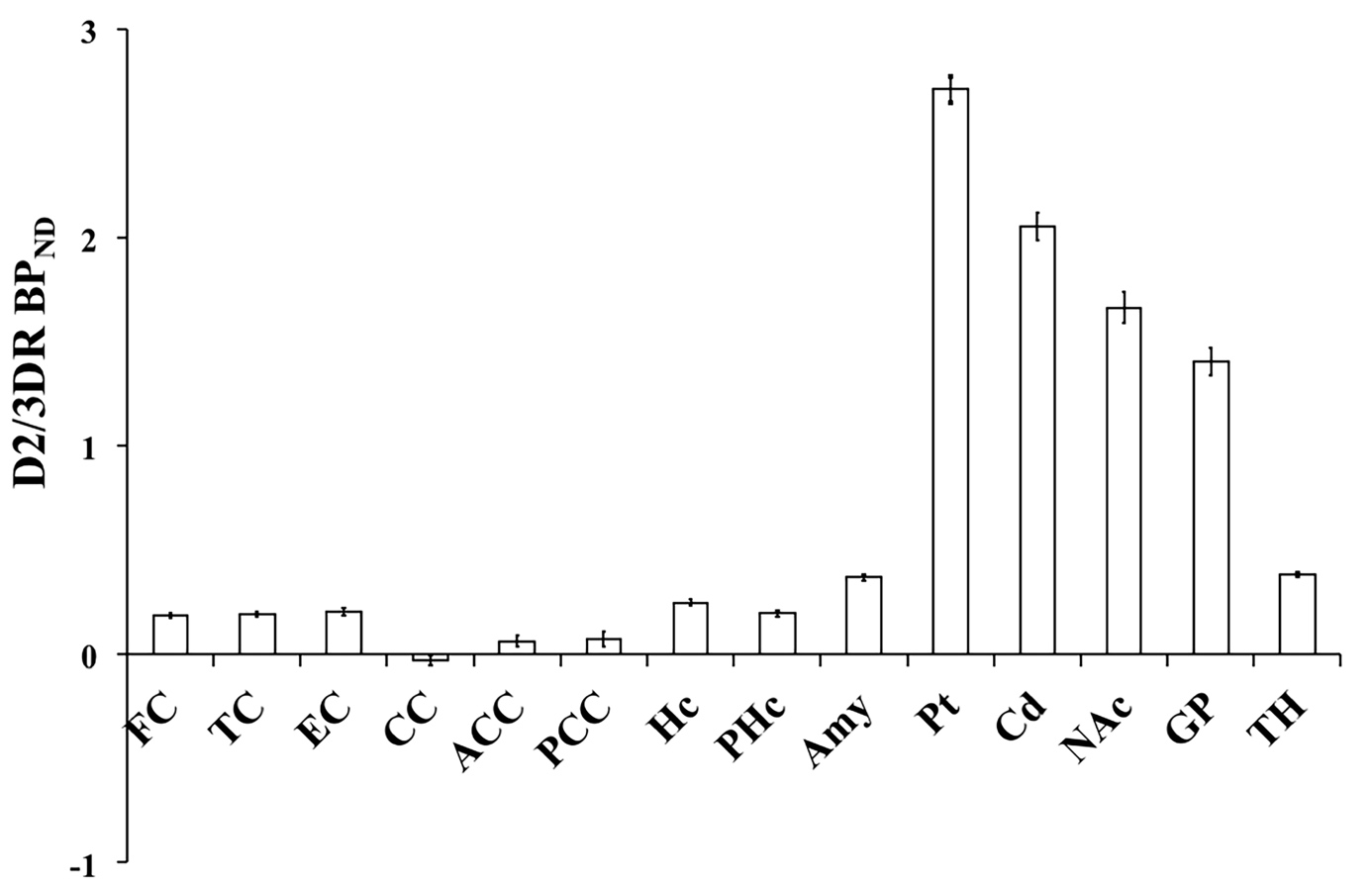
*

*Figure S2*. Mean [^11^C]raclopride D2/3DR BP_ND_ across brain regions using filtered back-projection reconstruction. FC = frontal cortex (superior, medial, orbital); TC = Temporal Cortex (superior, middle); EC = Entorhinal Cortex; CC = Corpus Callosum; ACC = Anterior Cingulate Cortex; PCC = Posterior Cingulate Cortex; Hc = Hippocampus; PHc = Parahippocampus; Amy = Amygdala; Pt = Putamen; Cd = Caudate; NAc = Nucleus Accumbens; GP = Globus Pallidus; TH = Thalamus. Error bars represent 95% confidence intervals around the means.

*
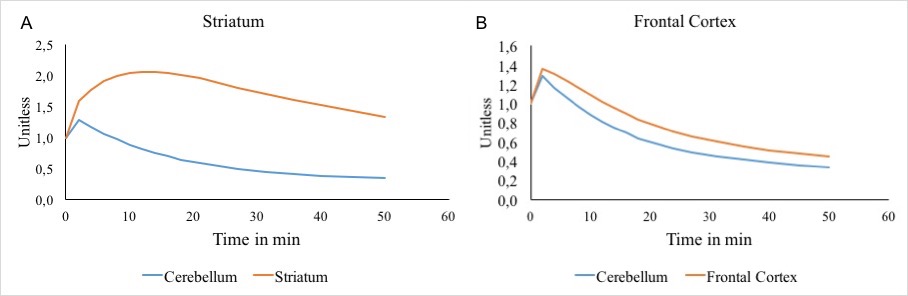
*

*Figure S3.* Time-activity-curves (TACs) for (A) striatum and (B) frontal cortex normalized to the first frame. A: The grey matter in cerebellum (blue) displays an exponential decay typical for a one-compartment model devoid of receptor binding. Striatum (red; average of putamen and caudate) displays a convex shape clearly visible in the first 25 minutes, which can only be explained by a two-compartmental model including receptor binding. B: Average of cortical region-of-interest (caudal middle frontal, lateral orbitofrontal, medial orbitofrontal, rostral middle frontal, superior frontal, frontal pole). The cortex also displays a convex shape in the first 25 minutes, which also requires a two-compartmental model and thus indicates receptor availability.

*
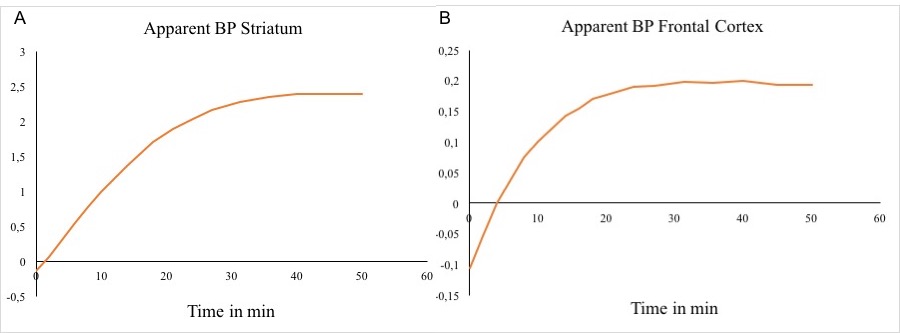
*

*Figure S4.* Illustration of apparent BP asymptotically reaching an equilibrium at later times, both for striatum (A) and frontal cortex (B).

The following ratio is plotted across time: (Region-of-interest – Cerebellum)/Cerebellum. Note that if the region-of-interest has the same kinetics as the cerebellum, the graphs should be a horizontal line at y=0. This is not the case for the frontal cortex (B), which behaves similar to the striatum (A).

*Table S1.* Correlations among select regions of interest derived from the iterative construction method (corresponding filtered-back projection data are given in parentheses).

|  | Pt R | Hc L | Hc R | FC L | FC R | PC L | PC R |
| --- | --- | --- | --- | --- | --- | --- | --- |
| Pt L | .84 (.85) | .20 (.29) | .17 (.19) | .24 (.24) | .23 (.29) | .18 (.19) | .17 (.20) |
| Pt R |  | .29 (.33) | .27 (.24) | .22 (.26) | .20 (.27) | .16 (.20) | .15 (.23) |
| Hc L |  |  | .81 (.74) | .46 (.51) | .47 (.51) | .55 (.54) | .52 (.53) |
| Hc R |  |  |  | .46 (.48) | .47 (.52) | .51 (.54) | .48 (.51) |
| FC L |  |  |  |  | .93 (.90) | .71 (.64) | .70 (.69) |
| FC R |  |  |  |  |  | .72 (.68) | .74 (.71) |
| PC L |  |  |  |  |  |  | .93 (.94) |

*Note*. All correlations above .15 are significant at *p*=.05. Pt = Putamen; Hc = Hippocampus; FC = Frontal Cortex; PC = Parietal Cortex; L = Left Hemisphere; R = Right Hemisphere.

*Table S2.* Extracted Bmax and Kd values from Hall et al. (1994).

| Region of interest | Bmax | Kd |
| --- | --- | --- |
| Frontal cortex, superior | 1.590 | 0.51 |
| Frontal cortex, medial | 0.276 | 0.96 |
| Frontal cortex, orbital | 0.490 | 0.48 |
| Temporal cortex, superior | 0.395 | 0.48 |
| Temporal cortex, medial | 0.753 | 1.76 |
| Entorhinal cortex | 0.326 | 1.11 |
| Cinguli anterior | 0.334 | 0.85 |
| Cinguli posterior | 0.349 | 0.68 |
| Parahippocampus | 0.225 | 0.22 |
| Hippocampus | 0.618 | 1.83 |
| Amygdala | 0.560 | 1.14 |
| Putamen, average | 10.16 | 1.32 |
| Caudatus, average | 6.98 | 1.17 |
| Accumbens | 8.007 | 1.42 |
| Globus pallidus | 2.278 | 1.48 |
| Thalamus | 0.747 | 2.86 |

*Table S3.* Mean [^11^C]raclopride D2/3DR BP_ND_ across brain regions.

| ROI | Mean | Lower 95% CI | Upper 95% CI |
| --- | --- | --- | --- |
| FC | .223 | .217 | .229 |
| TC | .253 | .247 | .259 |
| EC | .232 | .224 | .241 |
| CC | .-.023 | .-.034 | -.013 |
| ACC | .112 | .099 | .125 |
| PCC | .137 | .121 | .153 |
| Hc | .261 | .254 | .268 |
| PHc | .203 | .196 | .210 |
| Amy | .397 | .389 | .405 |
| Pt | 3.16 | 3.12 | 3.20 |
| Cd | 2.26 | 2.22 | 2.30 |
| NAc | 1.89 | 1.84 | 1.93 |
| GP | 1.42 | 1.38 | 1.45 |
| TH | .482 | .474 | .489 |

*Note*. FC = Frontal Cortex (superior, medial, oribital); TC = Temporal Cortex (superior, middle); EC = Entorhinal Cortex; CC = Corpus Callosum; ACC = Anterior Cingulate Cortex; PCC = Posterior Cingulate Cortex; Hc = Hippocampus; PHc = Parahippocampus; Amy = Amygdala; Pt = Putamen; Cd = Caudate; NAc = Nucleus Accumbens; GP = Globus Pallidus; TH = Thalamus; CI = Confidence Interval.

*Table S4.* Correlations among indicators of the hierarchical factor model portraying the relationship between [^11^C]raclopride D2/3DR BP_ND_ in striatum, limbic system, and neocortex.

|  | Pt R | Cd L | Cd R | Amy L | Amy R | Hc L | Hc R | FC L | FC R | TC L | TC R | PC L | | PC R | OC L | OC R |
| --- | --- | --- | --- | --- | --- | --- | --- | --- | --- | --- | --- | --- | --- | --- | --- | --- |
| Pt L | .84 | .55 | .61 | .34 | .24 | .20 | .17 | .24 | .23 | .35 | .24 | .18 | | .17 | .25 | .26 |
| Pt R |  | .54 | .62 | .33 | .28 | .29 | .27 | .22 | .20 | .32 | .25 | .16 | | .15 | .20 | .18 |
| Cd L |  |  | .80 | .29 | .12 | .33 | .27 | .17 | .17 | .25 | .23 | .15 | | .19 | .20 | .18 |
| Cd R |  |  |  | .34 | .25 | .30 | .28 | .18 | .16 | .24 | .23 | .15 | .13 | | .21 | .18 |
| Amy L |  |  |  |  | .56 | .44 | .46 | .42 | .48 | .49 | .46 | .37 | | .37 | .42 | .41 |
| Amy R |  |  |  |  |  | .44 | .55 | .37 | .38 | .39 | .43 | .27 | | .25 | .35 | .28 |
| Hc L |  |  |  |  |  |  | .81 | .46 | .47 | .57 | .59 | .55 | | .52 | .59 | .54 |
| Hc R |  |  |  |  |  |  |  | .46 | .47 | .56 | .63 | .51 | | .48 | .59 | .48 |
| FC L |  |  |  |  |  |  |  |  | .93 | .71 | .67 | .71 | | .70 | .60 | .60 |
| FC R |  |  |  |  |  |  |  |  |  | .69 | .71 | .72 | | .74 | .63 | .61 |
| TC L |  |  |  |  |  |  |  |  |  |  | .85 | .60 | | .56 | .65 | .63 |
| TC R |  |  |  |  |  |  |  |  |  |  |  | .61 | | .62 | .66 | .61 |
| PC L |  |  |  |  |  |  |  |  |  |  |  |  | | .93 | .75 | .73 |
| PC R |  |  |  |  |  |  |  |  |  |  |  |  | |  | .73 | .73 |
| OC L |  |  |  |  |  |  |  |  |  |  |  |  | |  |  | .84 |

*Note*. All correlations above .15 are significant at *p*=.05. Pt = Putamen; Cd = Caudate; Amy = Amygdala; Hc = Hippocampus; FC = Frontal Cortex; TC= Temporal Cortex; PC = Parietal Cortex; OC = Occipital Cortex; L = Left Hemisphere; R = Right Hemisphere.

*Table S5.* Factor loadings of an alternative model positing that common variance across the indicators generalizes across striatum, limbic system, and neocortex.

| ROI | Left | Right |
| --- | --- | --- |
| Pt | .232 | .188 |
| Cd | .208 | .201 |
| Amy | .446 | .353 |
| Hc | .603 | .572 |
| FC | .821 | .836 |
| TC | .656 | .673 |
| PC | .948 | .943 |
| OC | .815 | .794 |

*Note*. ROI = Region of interest; Pt = Putamen; Cd = Caudate; Hc = Hippocampus; Amy = Amygdala; FC = Frontal Cortex; TC= Temporal Cortex; PC = Parietal Cortex; OC = Occipital Cortex; L = Left Hemisphere; R = Right Hemisphere.

*Table S6.* Correlations among indicators of the hierarchical factor model reflecting interrelations of [^11^C]raclopride D2/3DR BP_ND_ between functional subdivisions in extrastriatal regions.

|  | Hc  R | Amy  L | Amy R | OFC  L | OFC R | ACC  L | ACC R | BA9 L | BA9 R | BA46  L | BA46  R | Pre L | Pre R | Post L | Post R | SupPar L | SupPar R |
| --- | --- | --- | --- | --- | --- | --- | --- | --- | --- | --- | --- | --- | --- | --- | --- | --- | --- |
| Hc L | .81 | .44 | .44 | .45 | .45 | .47 | .50 | .14 | .12 | .27 | .26 | .44 | .47 | .40 | .43 | .26 | .38 |
| Hc R |  | .46 | .55 | .46 | .48 | .41 | .48 | .12 | .06 | .29 | .27 | .43 | .48 | .35 | .40 | .20 | .33 |
| Amy L |  |  | .56 | .40 | .40 | .41 | .43 | .13 | .18 | .18 | .24 | .32 | .37 | .24 | .32 | .21 | .27 |
| Amy R |  |  |  | .43 | .40 | .28 | .34 | -.01 | -.04 | .14 | .13 | .18 | .27 | .06 | .21 | .13 | .21 |
| OFC L |  |  |  |  | .90 | .53 | .55 | .05 | .02 | .16 | .20 | .36 | .36 | .30 | .29 | .23 | 26 |
| OFC R |  |  |  |  |  | .51 | .57 | .12 | .07 | .20 | .26 | .37 | .42 | .29 | .29 | .24 | .29 |
| ACC L |  |  |  |  |  |  | .81 | .51 | .51 | .52 | 52 | .61 | .63 | .52 | .52 | .35 | .33 |
| ACC R |  |  |  |  |  |  |  | .52 | .48 | .56 | .52 | .62 | .62 | .52 | .50 | .46 | .43 |
| BA9 L |  |  |  |  |  |  |  |  | .84 | .79 | .73 | .57 | .53 | .50 | .52 | .34 | .29 |
| BA9 R |  |  |  |  |  |  |  |  |  | .70 | .75 | .50 | .54 | .56 | .51 | .39 | .33 |
| BA46 L |  |  |  |  |  |  |  |  |  |  | .82 | .56 | 49 | .49 | .51 | .19 | .21 |
| BA46 R |  |  |  |  |  |  |  |  |  |  |  | .52 | .53 | .51 | .53 | .24 | .23 |
| Pre L |  |  |  |  |  |  |  |  |  |  |  |  | .80 | .79 | .70 | .53 | .59 |
| Pre R |  |  |  |  |  |  |  |  |  |  |  |  |  | .70 | .80 | .61 | .65 |
| Post L |  |  |  |  |  |  |  |  |  |  |  |  |  |  | .75 | .56 | .60 |
| Post R |  |  |  |  |  |  |  |  |  |  |  |  |  |  |  | .56 | .57 |
| SupPar L |  |  |  |  |  |  |  |  |  |  |  |  |  |  |  |  | .89 |

*Note.* All correlations above .15 are significant at *p*=.05. Hc = Hippocampus; Amy = Amygdala; OFC = Orbito-Frontal Cortex (lateral, medial); ACC = Anterior Cingulate Cortex; BA9 = Brodmann Area 9; BA46 = Brodmann Area 46; Pre = Precentral Gyrus; Post = Postcentral Gyrus; SupPar = Superior Parietal Lobule; L = Left Hemisphere; R = Right Hemisphere.

*Table S7.* Factor loadings of an alternative model positing that common variance across the indicators generalizes across functional subdivisions in extrastriatal regions.

| ROI | Left | Right |
| --- | --- | --- |
| Hc | .591 | .563 |
| Amy | .464 | .348 |
| OFC | .533 | .559 |
| ACC | .782 | .811 |
| BA9 | .608 | .583 |
| BA46 | .606 | .614 |
| Precentral | .832 | .858 |
| Postcentral | .748 | .763 |
| Superiorparietal | .635 | .649 |

*Note*. ROI = Region of interest; Hc = Hippocampus; Amy = Amygdala; OFC = Orbito-Frontal Cortex (lateral, medial); ACC = Anterior Cingulate Cortex; BA9 = Brodmann Area 9; BA46 = Brodmann Area 46; Precentral = Precentral Gyrus; Postcentral = Postcentral Gyrus; Superiorparietal = Superiorparietal Lobule; L = Left Hemisphere; R = Right Hemisphere.
